# Early Failure of Total Ankle Arthroplasty in a Patient with Ipsilateral Varus Knee Deformity

**DOI:** 10.1155/2021/5245396

**Published:** 2021-09-24

**Authors:** Taehyeon Kim, Su Chan Lee, Chang Hyun Nam, Suengryol Ryu, Hye Sun Ahn, Ji-Hoon Baek

**Affiliations:** Joint & Arthritis Research, Himchan Hospital, Seoul, Republic of Korea

## Abstract

Multiple risk factors such as age, body mass index (BMI), preoperative diagnosis, smoking, diabetes mellitus, malalignment of an implant, and presence of ipsilateral hindfoot fusion have been shown to contribute to failure of total ankle arthroplasty (TAA). However, the exact causes of TAA failure remain uncertain, and various causes can lead to a need for revision surgery. We report a case of early aseptic loosening of the implant following TAA in a patient with severe varus deformity of the ipsilateral knee.

## 1. Introduction

Total ankle arthroplasty (TAA) is one of the surgical treatment options for end-stage osteoarthritis of the ankle, and it can decrease pain and improve function [1]. Ankle arthrodesis (AA) has been the gold standard for end-stage ankle osteoarthritis, but the popularity of TAA has increased rapidly due to promising results in recent years [2, 3]. However, complications and failure rates following TAA remain high compared with those for AA [4]. Although several studies have identified some predisposing factors associated with failure of TAA including age, sex, race, type of implant, malposition of implant, and radiologic findings [5, 6], the exact causes of early failure remain uncertain, and a variety of causes can result in need for revision.

We report a case of a 72-year-old male patient with concurrent osteoarthritis of the ankle and an ipsilateral varus knee in whom TAA was performed, resulting in early aseptic loosening of the implant.

## 2. Case Presentation

**Figure 1:**
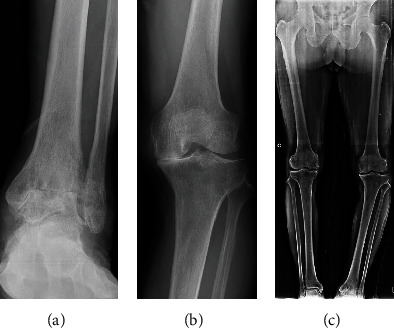
(a) An anteroposterior (AP) radiograph of a 72-year-old male patient who developed Takakura's classification stage 4 osteoarthritic changes of the left ankle that failed to respond to conservative management. (b) An AP radiograph of Kellgren-Lawrence Grade IV osteoarthritic changes in the left knee (c) A preoperative standing AP radiograph of the bilateral lower extremities showed a left hip-knee-ankle (HKA) angle of 14.3°.

A 72-year-old male patient who complained of serious pain in the left ankle and ipsilateral knee for about five years presented to the clinic in January 2017. He had no systemic musculoskeletal disease or other medical history such as hypertension or diabetes mellitus. He was 159.0 cm in height and 60.0 kg in weight, with a body mass index of 23.7 kg/m^2^. The patient reported difficulty walking more because of his left ankle pain than his left knee pain, and he had experienced no improvement of his symptoms after receiving medication and injection treatment at another clinic. The physical examination revealed a large effusion and pain with limited range of motion in the left ankle. The osteoarthritic stage of the ankle was Takakura's classification stage 4 [7] (Figure 1). In addition, the left knee was swollen and showed a 5° flexion contracture and a varus deformity. A Kellgren-Lawrence (K-L) grade IV osteoarthritic change of the left knee was documented (Figure 1). The Hospital for Special Surgery (HSS) score for the left knee was 65. A weight-bearing X-ray revealed a severe varus deformity, and the hip-knee-ankle (HKA) angle was 14.3° (Figure 1).

**Figure 2:**
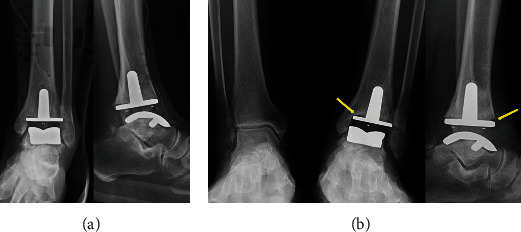
(a) The patient underwent total ankle arthroplasty (TAA). (b) Three months after TAA. The radiological assessment revealed a radiolucent line and osteolysis (yellow arrow) of the tibial component (standing view).

After considering all the findings, we decided to operate on the painful ankle first and then on the knee three months later. The patient underwent total ankle arthroplasty (TAA) with a Zenith Total Ankle Replacement (Corin, Cirencester, Gloucestershire, United Kingdom) (Figure 2(a)). The ankle was immobilized with a short leg splint for two weeks. After the splint was removed, the patient was advised to perform partial weight-bearing with a crutch after wearing protective boots for six weeks. Two months after the surgery, he initiated full weight bearing without a crutch and protective boots.

At three months following the procedure, the patient complained of medial side pain in the left ankle, especially on walking. Clinical examination revealed restriction of movement due to pain and tenderness on the medial side and edema of the ankle. Laboratory studies including a complete blood-cell and differential count, erythrocyte sedimentation rate (ESR), and C-reactive protein (CRP) level were within normal limits, and there was no clinical evidence of infection. Plain radiographs demonstrated a radiolucent line and osteolysis of the medial tibial component (Figure 2(b)). It was decided to prescribe medication and observe progress because the pain was not severe.

**Figure 3:**
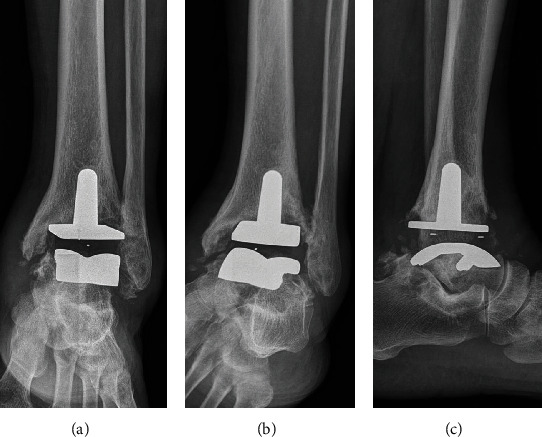
Six months after the TAA. (a, b) The radiological assessment revealed a progressive radiolucent line and osteolysis of the tibial component. (c) A lateral radiograph showed subsidence and a continuous radiolucency around the entire talar component.

**Figure 4:**
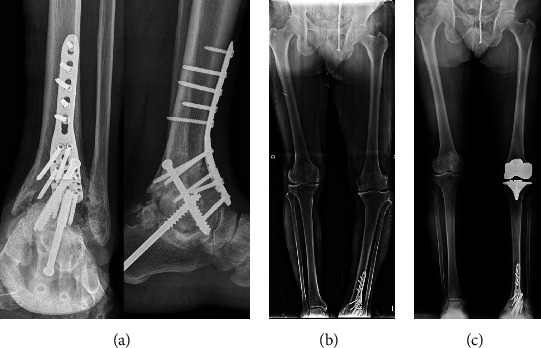
(a, b) The patient underwent tibiotalocalcaneal arthrodesis surgery. (c) Six months after tibiotalocalcaneal arthrodesis surgery, he underwent left total knee arthroplasty (TKA). A standing AP radiograph of the bilateral lower extremities revealed a 1.1° left HKA angle.

At six months postoperative, the patient complained of global pain in the left ankle joint, as well as tenderness and swelling. He reported aggravation of pain when his full body weight was loaded on his left ankle. Plain radiographs demonstrated a progressive radiolucent line and osteolysis of the tibial component. The talar component was not well positioned in the anteroposterior (AP) plane, sitting lateral to the midline (Figure 3). It was decided that ankle arthroplasty should be converted to an arthrodesis. The patient underwent tibiotalocalcaneal arthrodesis surgery (Figure 4(a)). He recovered without any complications and appeared to be doing well at three months postoperative follow-up. Six months after the ankle arthrodesis surgery, he underwent left total knee arthroplasty (TKA) and recovered without any complications (Figure 4(c)).

## 3. Discussion

The purpose of this report is to emphasize the need to evaluate the function and varus alignment of the knee joint both clinically and radiologically prior to TAA. In elderly patients with severe varus deformity of the knee, performing TKA first before TAA could be beneficial in reduction of the burden on ankle joints.

Numerous studies have reported the role of individual patient characteristics and clinical and radiographic factors as risk factors associated with failure of TAA. Such risk factors include age, body mass index (BMI), preoperative diagnosis, smoking, diabetes mellitus, malalignment of the implant, and presence of an ipsilateral hindfoot fusion. Patient age at the time of surgery was predictive of implant failure, and multiple authors have shown a relationship between younger patients and higher failure risk [8, 9]. Schipper et al. [10] found a significantly increased risk for early failure among patients with elevated BMI. LaMothe et al. [11] reported that patients with preoperative rheumatoid arthritis were at increased risk of failure within three months. Few studies have investigated the effects of smoking and diabetes on TAA failure [12, 13]. Multiple investigators have reported malalignment of TAA components as another likely risk factor for implant failure [14, 15]. Lewis et al. [16] found that ipsilateral hindfoot fusion increased the risk of failure after TAA. In our case, the patient was not associated with the above-mentioned risk factors. However, the possibility of catastrophic failure of implants and surrounding bones of the tibia and talus cannot be ignored. At three months after surgery, the patient complained of medial side pain in the left ankle on walking, and a radiolucent line and osteolysis of the medial tibial component were detected. At six months postoperative, the patient had difficulty walking because of his left ankle pain, and radiological assessment revealed evidence of component migration and aseptic loosening. Considering the above circumstances, we assumed that varus alignment in the lower extremity might be related to imbalance in weight distribution and related progression of implant loosening.

The changes in alignment in the proximal tibia can affect the loading conditions within the ankle joint [17]. Therefore, progressive varus alignment of the lower limb could result in a linear increase in tibiotalar contact area. Patients who suffer from end-stage osteoarthritis in both knee and ankle joints on the ipsilateral limb are encountered in clinical practice. There is no clear indication as to which to operate on first. However, it is reasonable to expect that the patient undergoing TAA might experience early loosening of the implant due to an imbalance in weight distribution from varus alignment in the lower extremity.

Our report represents a case of early failure in a patient with severe varus deformity of the knee following TAA. It demonstrates that it is possible to incorrectly distribute weight loading on the ankle in patients with ipsilateral varus knee deformity, and this might be a cause of early failure. It seems reasonable to evaluate the function and varus alignment of the knee joint both clinically and radiologically prior to TAA.
